# Desmoid Fibromatosis of the Anterior Abdominal Wall in Pregnancy: A Case Report and Review of the Literature

**DOI:** 10.3390/diseases12010027

**Published:** 2024-01-17

**Authors:** Pavol Zubor, Caroline Marie Henriksen, Maren Elvenes Økstad, Erika Cerskuviene, Jozef Visnovsky, Karol Kajo, Andrey Valkov, Kristen Olav Lind

**Affiliations:** 1 Department of Obstetrics and Gynecology, Nordland Hospital, 8450 Stokmarknes, Norway; 2 OBGY Health & Care Ltd., 01001 Zilina, Slovakia; 3 Faculty of Health Care, Catholic University, 03401 Ruzomberok, Slovakia; 4 VISNOVSKI Ltd., 03601 Martin, Slovakia; 5 Department of Pathology, St. Elisabeth Cancer Institute, 81250 Bratislava, Slovakia; karol.kajo1@gmail.com; 6 Department of Clinical Pathology, University Hospital of Northern Norway, 9019 Tromsø, Norway; andrej.yurjevic.valkov@unn.no

**Keywords:** desmoid tumor, benign neoplasm, chemotherapy, high-risk pregnancy, delivery, uncommon complications, abdominal discomfort, management

## Abstract

A desmoid tumor (DT) is a rare benign neoplasm arising from muscle aponeurosis, associated mostly with trauma or pregnancy. DT has an infiltrative and locally aggressive growth pattern and usually does not metastasize. However, it has a high recurrence and complication rate. When it occurs in pregnancy, the pregnancy and delivery is taken as an individual case for optimal management by physicians and midwifes, who need to be cautious in finding the optimal delivery mode for the patient, which depends on the tumor size, location, behavior, and past history. The authors report a case of 29-year-old pregnant woman who previously underwent systemic oncological treatment for a large abdominal wall desmoid tumor and became pregnant afterwards. The history of DT presented a follow-up and delivery challenge. Observational management was chosen with an elective cesarean section at week 38 + 4 of pregnancy with uncomplicated postpartum follow-up. The authors detail the clinical management and chosen therapeutic approach; chemotherapy can be a choice in the treatment options for patients with DTs, although the majority of DTs are treated surgically with subsequent mesh plastic. Moreover, the authors provide a systematic review of the literature focused on the treatment management of DTs in pregnant women during pregnancy and the postpartum period, as pregnancy-associated desmoid tumors are a specific condition, where the optimal management is not well established, despite some guidelines for non-pregnant patients.

## 1. Introduction

Desmoid fibromatosis (DF), sometimes named a desmoid tumor (DT), represents a rare type of tumor, accounting for 0.03% of all neoplasms and less than 3% of tumors arising from soft tissue [1]. DF, according to WHO (2020), is defined as a locally aggressive but non-metastasizing deep-seated (myo)fibroblastic monoclonal neoplasm with an infiltrative growth pattern and a tendency for local recurrence [2]. 

The incidence of DF is 2.4 to 5 per one million population per year. DTs predominantly affect young females, mostly between the ages of 30 and 40 years, and occurs more than twice as often in female than male patients [3,4,5]. 

Reflecting their location, desmoid tumors can be classified as extra-abdominal and abdominal. Abdominal DTs are either superficial (abdominal wall) or intra-abdominal. The extra-abdominal lesions mostly occur in the neck, shoulder, chest wall, breast, back, arm, buttock, thigh, and leg. Multicentric extra-abdominal desmoids are very rare, and they have specific clinical presentation [6,7]. 

Historically, these tumors were first described by MacFarland in 1832. Etymologically, the word “desmoid” was first used by Johannes Müller (1801–1858) in his monograph on cancer [8]. The name is derived from the Greek word «Desmos», which means band- or tendon-like [9]. In 1951, Gardner first described the common presence of desmoid tumors in patients with familial adenomatous polyposis (FAP).

The detailed pathogenesis and factors affecting the clinical behavior of DTs are still uncertain. However, most cases of DTs have been described in patients with previous abdominal trauma or surgery (either laparoscopic, robotic, or open) [10,11,12,13]. The management is multidisciplinary and often repetitive, as DTs are locally invasive and prone to high local recurrence after resection.

In this paper, we report a case of young female who was diagnosed with a desmoid tumor before pregnancy, underwent oncological treatment, and during pregnant developed a residual desmoid tumor in the anterior abdominal wall, thus leading to a special obstetric problem requiring individual management. Moreover, we are presenting an overview of the previously published cases where desmoid fibromatosis was associated with pregnancy. 

## 2. Case Report

A 29-year-old primiparous woman with physiological pregnancy was referred to a primary care hospital for follow-up during pregnancy and planning of the delivery with the first check-in at 12 + 4 weeks of pregnancy. Her past medical history was significant for FAP for which she underwent laparoscopic total proctocolectomy with ileoanal reservoir, with a transient right-sided relieving colostomy at the age of nineteen. This was due to papillous tubulovillious adenoma with low grade intraepithelial (LB-IEN) neoplasia. 

**Figure 1:**
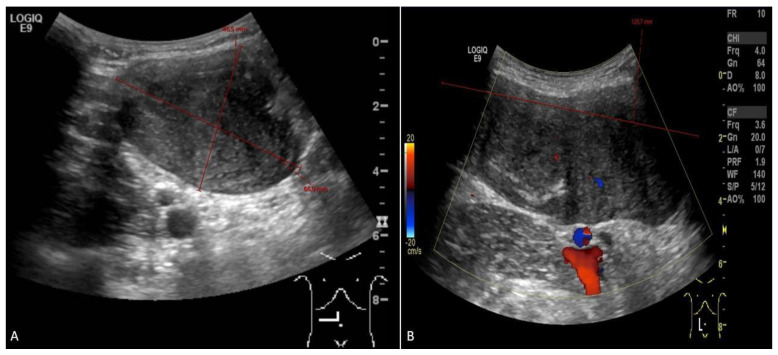
Abdominal ultrasound ((**A**)—transverse view; (**B**)—longitudinal view) picture of desmoid tumor, sized 12.0 × 4.6 × 6.6 cm on the first diagnosis.

**Figure 2:**
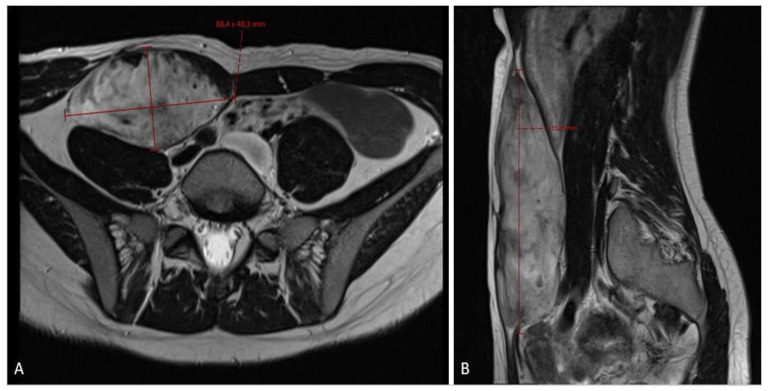
MRI scan ((**A**)—axial plane; (**B**)—sagittal plane) assessment of fibromatosis lesion at the first diagnostic scan, sized 8.8 × 4.8 × 15.3 cm.

**Figure 3:**
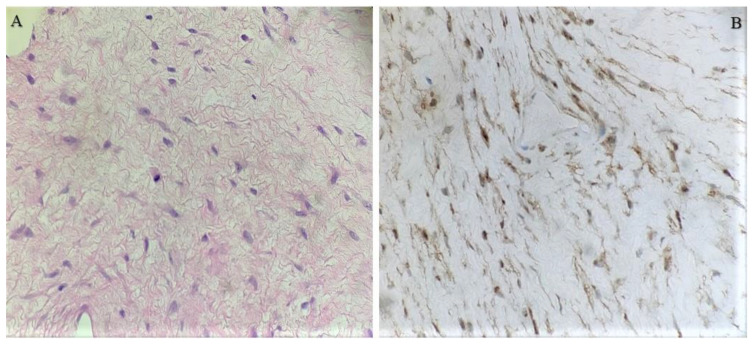
Histology of the desmoid fibromatous tumor with hematoxylin & eosin staining (**A**) and with the β-Catenin expression immunostaining (**B**).

When the patient turned 26 (6 years after the surgery), she developed a desmoid tumor sized 12.0 × 4.6 × 6.6 cm on abdominal ultrasound scan (Figure 1) and 8.8 × 4.8 × 15.3 cm on MRI scan (Figure 2) in the anterior abdominal wall on the right side close to the previous trocar point of incision after laparoscopic surgery for FAP and transient-relieving stomy. This was primarily suspected to be sarcoma or a desmoid tumor. A core-needle biopsy was performed confirming the diagnosis of desmoid fibromatous tumor (Figure 3). The tumor board considered the situation as stable, with the possibility for partial spontaneous regression. As the risk for recurrence was assessed to be 50%, no surgery was performed, and she was referred for follow-up with new MRI control in about 3 months. During this follow-up period, she became pregnant and underwent a provoked medical legal abortion in week 6. 

**Figure 4:**
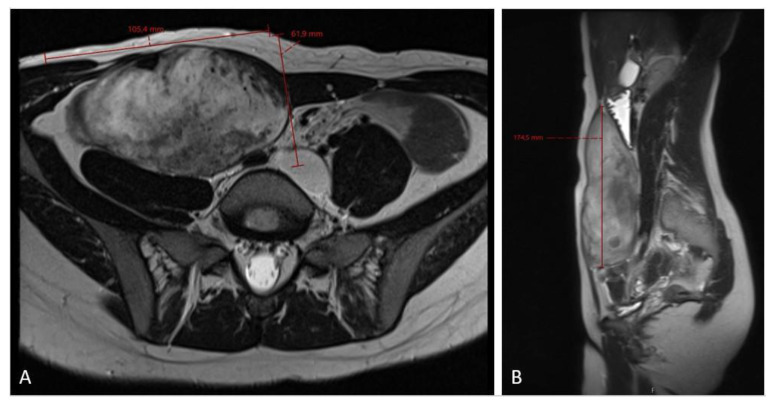
An MRI scan ((**A**)—axial plane; (**B**)—sagittal plane) at the 6-month follow-up checkup showing tumor growth progression, sized 10.5 × 6.2 × 17.5 cm in craniocaudal diameter, located in the musculus rectus abdominis on the right side.

**Figure 5:**
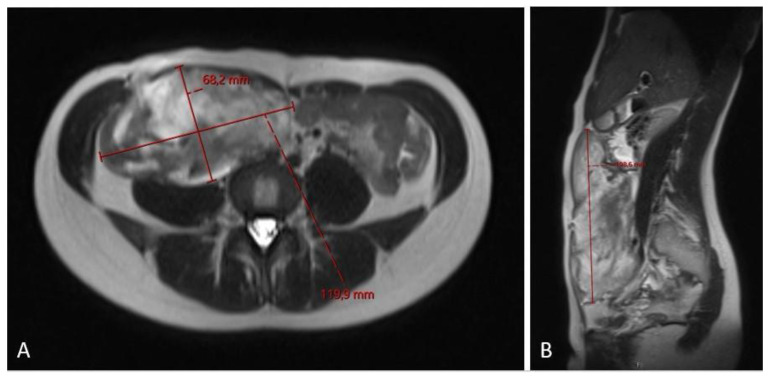
MRI scan ((**A**)—axial plane; (**B**)—sagittal plane) after 3 cycles of chemotherapy showing no effect of the treatment and slight progression in the craniocaudal tumor size (12.0 × 6.6 × 20.0 cm).

**Figure 6:**
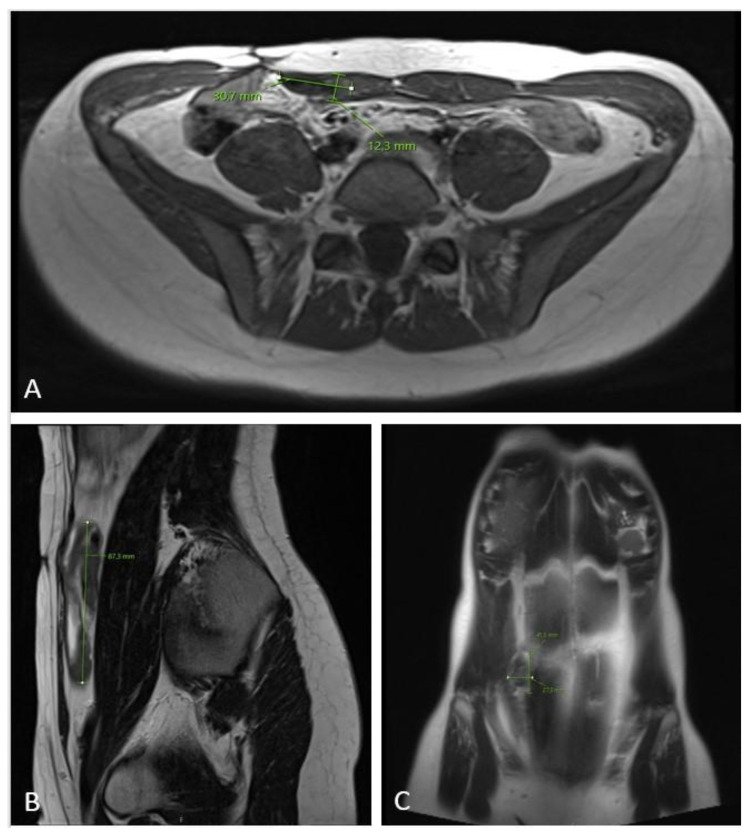
MRI scan ((**A**)—axial plane; (**B**)—sagittal plane; (**C**)—coronal plane) 12 months after chemotherapy showing significant tumor regress, sized only 1.2 × 3.7 × 8.7 cm.

The 3-month MRI control showed a stable tumor size; however, the MRI check at the 6-month follow-up showed significant tumor growth progression (Figure 4), sized 10.5 × 6.2 × 17.5 cm in craniocaudal diameter located in the musculus rectus abdominis on the right side. She was recommended to stop oral contraceptives and preventive devices (IUD) containing estrogen and/or progesterone and was referred to the National Oncology Center for further treatment, where the tumor board determined to administer chemotherapy, and the treatment started with three cycles of Caelyx (doxorubicin hydrochloride), 40 mg/m^2^ every fourth week, with MRI control afterwards. The patient suffered side effects in form of skin rashes, allergic respiratory problems, and mucositis. She received 60 mg of Caelyx by IV each cycle. The control MRI scan showed no effect of treatment; furthermore, there was a slight progression in the craniocaudal tumor size (12.0 × 6.6 × 20.0 cm), (Figure 5). As the oncologist awaited the latest response to chemotherapy, the further plan was to continue the same treatment options (Caelyx, although at a reduced dose, set to 30 mg/m^2^) due to the previous side effects. The patient underwent three further cycles with total dose of 47 mg doxorubicin at each cycle. The MRI scan after six cycles showed partial regression of the tumor, now sized 11.2 × 5.4 × 19.1 cm. She was continuously monitored by the surgeon and radiologist with MR scans every 4 months, and the 12-month scan after chemotherapy showed significant tumor regress, sized only 1.2 × 3.7 × 8.7 cm (Figure 6). Five weeks later, the patient became pregnant and was referred from the midwife for gynecological control, starting on week 12 + 4.

All the prenatal controls showed no worsening respiratory or bowels conditions and normal fetal growth charts over the pregnancy. Due to the previous total colectomy for FAP, chemotherapy and location of the tumor with size progression, the conclusion for an elective cesarean section (CS) was reached, and she delivered at 38 + 4 week of pregnancy through uncomplicated CS with Pfannenstiel incision and low transverse corporal incision on the uterus. She delivered the healthy baby weighing 3435 g, with a length of 48 cm and an Apgar score 10-10-10. The postpartum period was uncomplicated. Follow-up correspondence was conducted at 4 and 10 months postpartum, and the patient had recovered well, with a normal physical activity in life, the DT remained stable in size, and the patient is followed-up by regular checkups by gastric surgeons for the desmoid tumor and FAP with regular colonoscopies and MR scans. 

## 3. Discussion

DT histologically arises from the connective tissues of muscle (the result of abnormal monoclonal fibroblastic proliferation or the proliferation of myofibroblasts), the fascia, or the aponeurosis. The studies focused on the pathogenesis of these tumors showed the important role of impairments in the Wnt/β-catenin signaling pathway or mutations in the *APC* and *CTNNB1* genes as a ‘key’ trigger for the development of desmoid tumors [14,15]. Furthermore, the analysis of the patomechanism of desmoid tumors showed that 90% of them are sporadic, and the remaining 5–10% display a genetic link to FAP (Gardner syndrome). Patients with this syndrome have 800–1000 times greater risk of developing DTs than the general population [15,16]. 

As for the topical anatomic location, DT can be present in extra-abdominal locations (49%), the abdominal wall (40%), and intra-abdominal areas, including the retroperitoneal space (8%) [5]. Sporadic DTs arise most commonly in extra-abdominal areas, but those related to FAP are generally found within the small bowel mesentery and/or in the abdominal wall, predominantly arising in soft tissue [17,18,19,20], causing bowel obstruction or ulceration and ureter stenosis. Patients with FAP often develop intra-abdominal tumors after abdominal trauma or surgery [2]. Moreover, the risk of DTs in FAP is also increased by certain pathologies in APC variants [21].

On gross macroscopy, DT resembles scar tissue with a firm consistency and gray or whitish color. Under light microscopy, it appears as a heterogeneous poorly characterized and uniform proliferation of spindle cells that resembles myofibroblasts wrapped inside a stroma of abundant collagen and a vascular network missing capsule. No necrosis, cellular atypia, or increased mitotic activity is noted. Inside the nuclei, there may be euchromatin or heterochromatin detected. There is no histological difference between sporadic DT and FAP-related DT; however, their molecular profile can be different. On immunohistochemistry (IHC), DTs are characterized by nuclear positivity for β-catenin, which reflects the activation of the WNT signaling pathway and is crucial for the diagnosis. Other markers often showing positivity include smooth muscle actin, muscle-specific actin, vimentin, PDGFRb, COX-2, androgen, and β-estrogen receptors. DTs are essentially negative for S-100, h-caldesmon, desmin, C-KIT, and CD34 [2,15,22]. Apart from the simple diagnostic aim of these IHC analyses, recent studies focused on clinicopathological assessment through determining the expression status of the programmed death-1/programmed death ligand 1 (PD1/PD-L1) immune checkpoint mechanism in correlation with β-catenin and CD4, together with other immune molecules (e.g., IL-2, IFN-ɤ, and CD8), showed promising results pointing out that PD-L1-centered immune checkpoint mechanisms may be involved in the tumor microenvironment of DTs [23] and linked to DT prognosis. Thus, they urge its inclusion in the diagnostic algorithms. 

Although histology is the gold standard for diagnosis, imaging modalities represent the basic tool in the diagnostic process of these tumors. To establish an adequate therapeutic approach, the proper diagnosis is necessary. Multimodal imaging tools, including computed tomography (CT), ultrasound (US), and magnetic resonance imaging (MRI), are helpful tools in the assessment process. These techniques can be also used to guide minimally invasive interventions and monitor their effectiveness in treatment [24]. 

The biological course of desmoid fibromatosis in certain patients varies a lot. These tumors show a high recurrence rate even when they are benign upon microscopic histological assessment. Their biologic behavior often indicates patterns of a “malignant” disease because the severe progression and local infiltration in vital organs leads to their impairment and sometimes to patient death. Certain clinical presentations may vary from asymptomatic lesions to impairing tumors with unpredictable growth, stabilization, and even regression. The variety of symptoms is directly associated with the size, location and progression speed of DTs. 

Intra-abdominal DTs grow asymptomatically until they reach large dimensions, leading to intestinal, urinary, vessel obstruction, or tissue ischemic damage with possible perforations or bleeding [25,26]. These effects on the surrounding organs and vessels may greatly complicate the surgical treatment [27,28]. On the other hand, a subset of DTs may exhibit spontaneous regression [29]. Rarely is complete remission seen, despite the recurrent feature of DTs after only simple observation [30].

Since their clinical behavior is heterogeneous and unpredictable, where the outcome is impacted by the anatomic area, proximity to crucial organs, affiliation with FAP, and natural behavior, the treatment ought to be individualized to decrease the risk for treatment failure with subsequent tumor recurrence and to achieve an acceptable morbidity with the highest possible quality of life [31]. Numerous issues with respect to the ideal treatment of desmoids remain in dispute. In any case, wide surgical extraction remains the treatment of choice, except when it is mutilating or is associated with significant organ function impairment, disabling chronic symptoms, and morbidity [15]. However, the involvement of surgical margins is related to an increased risk of local recurrence. Roughly 25–60% of patients show recurrence after resection. In this case, surgical re-resection, adjuvant radiation, systemic therapy, or close clinical follow-up might all be appropriate alternatives [32]. 

For these reasons, a watchful waiting approach with an individual period of initial observation has been advised for asymptomatic patients [33]. Point-by-point physical examination, imaging by ultrasound, CT, and MRI, and if not previously conducted, biopsy should be performed in accordance with the recommendations for soft tissue sarcomas as adopted at the consensus meeting in Milan (Italy, June 2018) [34]. Following this consensus, the current strategy for management of DT advocates for an «active surveillance» period. This approach does not seem to impact the efficacy of the ensuing treatment when required but permits the clinician to arrange the following step in the therapeutic management.

Apart from a surgical approach, in recent years, there has been increasing evidence proving significant progression in systemic therapeutic options (nonsteroidal anti-inflammatory drugs, anti-hormonal agents, tyrosine kinase inhibitors, low-dose chemotherapeutic regiments, and conventional chemotherapy as anthracycline-based regimens, mostly liposomal doxorubicin-/as described in our case/), immunotherapy, and biological or focused technological treatment (e.g., cryoablation, high-intensity focused ultrasound, and/or adjuvant radiotherapy) for these patients [35]. 

There have been around 100–120 cases of DTs in pregnancy worldwide reported, as published on PubMed, however only a few cases with systemic therapy for DT. The commonality in these reports was the strong biological variability of DTs depending on the hormonal background and the mostly watch-and-see management with close follow-up and operative delivery. Moreover, the reports describing DTs in pregnancy showed that the clinical picture of DTs can be unpredictable, especially when they develop before delivery or in the postpartum period. Most of the cases initially diagnosed in pregnancy were biopsied (core biopsy), and if malignity was excluded, they were managed conservatively by follow-up with precise MRI scans for the assessment of the DT size and location, where pregnancy was ended by CS with DT resection during CS or in the postpartum period (Table 1) [26,30,36,37,38,39,40,41,42,43,44,45,46].

For decades, DF has been thought to be a conceivable hormone-dependent malignancy based on the following arguments: estrogen receptors’ positivity on immunohistological assessment, the predominance of female patients with high incidence in the fertile age, studies reporting the diagnosis or relapse of a desmoid tumor in and/or after pregnancy, and observed tumor shrinkage with the administration of anti-estrogen drugs or in the postpartum period.

One of the first multicentric studies focusing on DT behavior and pregnancy was published in Italy by Fiore et al., 2014, [29], based on ninety-two women with DT; the authors analyzed the long-term data linked to the disease presentation during and after pregnancy. They showed that the initial treatment of DT in pregnant women was resection (52%), watchful waiting (43%), and later progression in 63%; only 4% of patients received medical therapy. Only 13% of patients relapsed after surgery. After pregnancy, 46% underwent treatment for DT, whereas 54% were managed with watchful waiting. Only 17% showed further progression after treatment. Spontaneous regression occurred in 14%. After further pregnancies, only 27% of patients progressed. The only adverse obstetric event was a higher rate of cesarean sections [29]. The linkage to pregnancy and postpartum events was further strengthened by the report from Hanna et al., who reported a case of rapidly progressing DT in the postpartum period [39], which was recently also confirmed by Debaudringhien et al., who showed that a history of pregnancy was associated with an increased risk of progression/relapse in patients with newly diagnosed DF, whereas hormonal contraception did not show a connection with disease progression or relapse [47]. 

Important knowledge in the management of DTs in pregnancy was introduced by Cates et al., who found that pregnancy itself does not increase the risk for local recurrence after the surgical resection of desmoid tumors [48]. Based on these findings, it seems that pregnancy can be taken as a risk factor for promoting the growth and progression of desmoid-type fibromatosis in ongoing pregnancy and postpartum because of the estrogenic stimulation of desmoid growth; however, it has a low impact on the recurrence of a DT after its surgical resection. Thus, subsequent pregnancy should not be discouraged for women at a fertile age after the surgical resection of desmoid tumors.

## 4. Conclusions

Pregnancy-associated DTs are rare, and the optimal management of these tumors is yet to be established. Nowadays, controversy centers on the follow-up approach or proper timing of surgical resection, which may be influenced by the increased potential for tumor growth and the negative reactions of a gravid uterus. Surgical resection of these tumors has been performed successfully both during and early after delivery, where postpartum radiotherapy, chemotherapy, and other medical interventions showed their effectiveness. The risk for disease progression during pregnancy is high; but, it can be safely managed. Pregnancy-related DF has good outcomes. As desmoid tumors do not significantly increase the obstetric risk, they cannot be a contraindication for future pregnancies. 

The management of desmoid tumors, especially when diagnosed during pregnancy is complex and requires multidisciplinary expertise by an experienced team where treatment has to be individualized. We must identify the reliable prognostic/predictive factors, as they are the key to assessing the efficacy of local or systemic treatment. A better understanding of the genetics and molecular alterations in signaling pathways has enriched this attitude and has led to the development of tailored interventions for DT, which could revolutionize its therapy and management strategies.

## Figures and Tables

**Table 1:** An overview on selected previously published cases of desmoid tumors in pregnancy showing variable clinical behaviour and different approaches in management of DT. (LPT—laparotomy; LSC—laparoscopy; CS—caesarean section; DT—desmoid tumor; N/A—not available; g.w.—gestational week; g—gram).

Reference	Patient Age (Years)	Parity	Time at Diagnosis	Previous Surgery	History of FAP/Gardner’s Syndrome	Tumor Location	Initial Tumor Size (cm)	Tumor Size at Delivery (cm)	Management and Treatment
Mohd Sulaiman et al., 2022 [26]	20	Para 1	13th g.w.	SC	Yes	Left rectus abdominis Right hypochondrium	4 × 7 × 10 2 × 4 × 5	15 × 12 5 × 5	Follow-up, Elective CS at 34th g.w. due to progression and local compression urinary symptoms
Marsh-Armstrong et al., 2021 [36]	28	Para 1	Before pregnancy	N/A	No	Intraabdominal pelvic and right obturator fossa	13.3	27 × 15 × 8	Failed cryoablation during pregnancy, followed by 2 cycles of doxorubicin (75 mg/m^2^, 21-day cycles) Elective CS at 35th g.w. due to growth progression and local urinary and bowel compression symproms Radical surgery 6 months after delivery
Jin et al., 2020 [37]	28	Para 1	32nd g.w.	LSC total colectomy	Yes	Intra-abdominal, left side of the uterus	21 × 12	N/A	Follow-up, spontaneous vaginal delivery without complications Radical surgery at 1 month post partum
Palacios-Zertuche et al., 2017 [38]	28	Para 0	5th g.w.	N/A	No	Abdominal wall	11 × 15 × 18	30 × 24 × 6 (5680 g)	Follow-up, Elective CS at 39 th g.w. Laparotomy, with tumour resection, hysterectomy and left salpingo- oophorectomy post partum
Hanna et al., 2016 [39]	19	Para 0	34th g.w.	No	No	Intra-abdominal mass - distal small bowel mesentery	12	N/A	Follow-up, spontaneous vaginal delivery without complications Radical surgery for 30 cm × 24 cm × 16 cm growth progression at 3mnd post partum
Sueishi et al. [30]	21	N/A	5 years before pregnancy	No	N/A	Internal obturator muscle	8	N/A	2-times operated for DT, followed by 2 deliveries, Spontaneous regression of DT one year after the last delivery
Awwad et al., 2013 [40]	40	Para 1	20th g.w.	SC	N/A	Right abdominal wall	N/A	12 × 9.5 × 7 (457 g)	Follow-up, Elective CS at 39th g.w. With radical resection of the tumor
Choi et al., 2012 [41]	36	Para 1	Before pregnancy	No	No	Right rectus abdominis muscle	3 × 2	3 × 3 × 2	Follow-up, spontaneous delivery, Minor incision post partum
Durkin et al., 2011 [42]	29	Para 1	First trimester	No	No	Left rectus abdominis muscle	3.5 × 7.2	N/A	En bloc resection of DT in 22nd g.w. sized 18.5 × 15 × 9.0 with mesh repair Follow-up and vaginal delivery at 39th g.w.
Michopoulou et al., 2011 [43]	37	Para 2	16th g.w.	SC	No	Right anterior abdominal wall	3 × 2	20 × 16	Electiv CS at 38th g.w. Postpartum resection and reconstruction with mesh
Viriyaroj et al., 2009 [44]	17	Para 1	5th month of pregnancy	No	N/A	Low abdominal wall rectus abd. Muscle and pelvic floor muscle	20 × 20	28 × 21 × 18 (4900 g)	Follow-up, Elective CS and complete surgical excision after delivery
Gurluler et al., 2014 [45]	35	Para 2	Post partum	SC	N/A	Anterior abdominal wall, Right rectus muscle	10 × 5	26 × 12 × 6.5 (420 g)	Radical tumor extirpation with mesch reconstruction
Zhou et al., 2015 [46]	31	Para 1	21th g.w.	No	N/A	Abdominal wall	15 × 12	35 × 30 × 14 (7100 g)	Elective CS at 35th g.w. with extirpation and mesh reconstruction

## Data Availability

Data supporting reported results can be found in Norwegian patients journal system DIPS.
